# Multifunctional Ginger Nanofiber Hydrogels with Tunable Absorption: The Potential for Advanced Wound Dressing Applications

**DOI:** 10.1021/acs.biomac.1c00215

**Published:** 2021-07-13

**Authors:** Paula Squinca, Linn Berglund, Kristina Hanna, Jonathan Rakar, Johan Junker, Hazem Khalaf, Cristiane S. Farinas, Kristiina Oksman

**Affiliations:** † Division of Materials Science, Department of Engineering Sciences and Mathematics, Luleå University of Technology, SE-971 87 Luleå, Sweden; ‡ Center for Disaster Medicine and Traumatology, Department of Biomedical and Clinical Sciences, Linköping University, SE-581 85 Linköping, Sweden; § Cardiovascular Research Centre, School of Medical Sciences, Örebro University, SE-703 62 Örebro, Sweden; ∥ Embrapa Instrumentation, Rua XV de Novembro 1452, 13561-206 São Carlos, SP, Brazil; ⊥ Graduate Program of Chemical Engineering, Federal University of São Carlos, Rod. Washington Luís-km 235, 13565-905 São Carlos, SP, Brazil; # Mechanical & Industrial Engineering, University of Toronto, 5 King’s College Road, Toronto, Ontario M5S 3G8, Canada

## Abstract

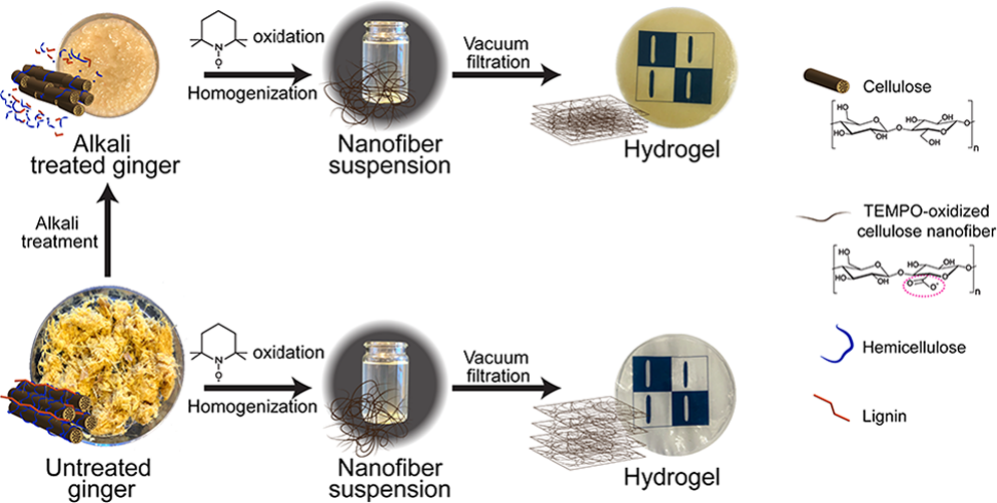

In this study, ginger residue from juice production was evaluated as a raw material resource for preparation of nanofiber hydrogels with multifunctional properties for advanced wound dressing applications. Alkali treatment was applied to adjust the chemical composition of ginger fibers followed by TEMPO (2,2,6,6-tetramethylpiperidine-1-oxyl radical)-mediated oxidation prior to nanofiber isolation. The effect of alkali treatment on hydrogel properties assembled through vacuum filtration without addition of any chemical cross-linker was evaluated. An outstanding absorption ability of 6200% combined with excellent mechanical properties, tensile strength of 2.1 ± 0.2 MPa, elastic modulus of 15.3 ± 0.3 MPa, and elongation at break of 25.1%, was achieved without alkali treatment. Furthermore, the absorption capacity was tunable by applying alkali treatment at different concentrations and by adjusting the hydrogel grammage. Cytocompatibility evaluation of the hydrogels showed no significant effect on human fibroblast proliferation in vitro. Ginger essential oil was used to functionalize the hydrogels by providing antimicrobial activity, furthering their potential as a multifunctional wound dressing.

## Introduction

Advanced wound dressings not only passively cover wounds but also provide functions that promote healing or improve wound care. An ideal wound dressing should provide a moist environment, absorb exudates, and protect against bacterial infection, while also being biocompatible and mechanically stable.^1,2^ It is generally accepted that a moist environment facilitates the healing of acute and chronic wounds^3−5^ and also facilitates epidermal cell migration, increasing the rate of re-epithelialization and angiogenesis.^6,7^

Hydrogels have been considered a suitable material for moist wound dressings since they absorb and retain water, which provides a moist environment for the wound. Hydrogels can have properties such as nonadhesion, biocompatibility, and transparency. These properties can be leveraged to develop advanced wound dressings with superior and novel functions, such as enabling continuous monitoring of the wound without removal of the dressing.^7−10^ The low absorbent capability of some hydrogels geared toward wound dressings has encouraged the development of new “superabsorbent” products that exhibit high degrees of swelling, often well over 10 times their dry mass.^11^ This property may be useful for high rates of wound exudate absorption, which may be important for wound dressing applications.^12^

The degree of hydration can change the mechanical properties of hydrogels, often resulting in worse mechanical stability in a swollen state. This can limit their practical application as wound dressings.^13^

2,2,6,6-Tetramethylpiperidine-1-oxyl (TEMPO)-oxidized cellulose nanofibers present attractive characteristics, such as small and uniform widths (around 3 nm) and high aspect ratio (higher than 150), that distinguish them from nonoxidized nanofibers that can provide transparent and strong network formation with increased mechanical performance.^14^ In recent years, nanofiber-based hydrogels have been exploited for wound dressing applications.^15−20^ However, to achieve the desired multifunctional properties, such as swelling combined with mechanical stability, the preparation procedures of these hydrogels are often complex involving cellulose nanofibers in combination with several different components such as alginate,^16^ chitosan,^17,19^ and cross-linking agents^16,19^ for the stability.

The use of silver nanoparticles in wound dressings is a well-known approach for minimizing microbial contamination of wounds and achieving antimicrobial materials.^15^

However, silver nanoparticles can be difficult to control and their use is known to have side effects on patient health.^21^ Biobased materials with novel, nature-derived antimicrobial properties have recently been explored as an important advanced functionalization of wound dressings.^22^ This approach can reduce the microbial challenge to wounds, thereby minimizing the risks associated with wound infections, all the while avoiding the further use of classical antibiotics, the use of which exasperates the threat of antimicrobial resistance.^22^

*Zingiber officinale* Roscoe, commonly known as ginger, is extensively used as a spice, but it has also been used as traditional medicine due to its purported antioxidant, antiviral, antidiabetic, anti-inflammatory, anticancer, as well as antibacterial properties.^23−27^ Ginger is rich in constituents such as cellulose, starch, and hemicellulose but also includes several bioactive families of compounds, such as gingerols, zingiberene, and shogaols.^28^ Ginger essential oil (GEO), mainly composed of geranial, α-zingiberene, (*E*,*E*)-α-farnesene, neral, and ar-curcumene, has also shown significant antimicrobial, antifungal, and antioxidant activities.^29,30^ Although the pharmacological properties have been supported by in vivo and in vitro experiments,^31^ only a few studies have focused on using its nanofibers for the preparation of bionanocomposites,^32−34^ films,^35−37^ and aerogels.^38,39^ The potential use of ginger and its natural components for nanofiber extraction and their utilization for the preparation of completely ginger-based hydrogels have, so far, not been studied for wound dressing applications.

In this study, we investigated the potential use of ginger for nanofiber extraction and subsequent assembly into hydrogels aimed at wound dressing applications. The hydrogels were prepared by simple vacuum-assisted filtration using only ginger nanofibers without any cross-linker to maintain low energy requirements, minimize the components needed for sustainable production, and avoid the risk of introducing toxic side effects. Alkali treatment was applied on ginger fibers before TEMPO oxidation to modify their chemical composition, altering the liquid absorption capacity of the hydrogels, which is evaluated in water, bovine serum albumin (BSA), and phosphate-buffered saline (PBS) solutions. The functionalization of ginger-based hydrogels with ginger essential oil seems like an advantageous strategy because it has the potential to leverage the antimicrobial properties of the plant to enhance the antimicrobial properties of the wound dressing while being sourced from the same raw materials. The structural morphology, mechanical properties in wet conditions, cytocompatibility, and antimicrobial properties of two versions of ginger nanofiber (GNF) hydrogels were quantified, supporting an initial evaluation of these materials for use in advanced wound dressing products.

## Experimental Section

### Materials

Ginger roots were purchased from a local market, and the fibers were obtained after juicing and used as the feedstock in this study. High-purity sodium chlorite (NaClO_2_), with a sodium chlorite content of 77.5–82.5% w/w, was purchased from VWR International AB (Stockholm, Sweden). Ginger essential oil, sodium hypochlorite (NaClO, 6–14% active chlorine), 2,2,6,6-tetramethylpiperidin-1-oxyl (TEMPO, 98%), bovine serum albumin (BSA) lyophilized, and phosphate-buffered saline (PBS) were purchased from Sigma-Aldrich, Sweden AB (Stockholm, Sweden). Glacial acetic acid (CH_3_COOH, 100%) was purchased from Merck KGaA (Darmstadt, Germany). BSA was used in a concentration of 50 g L^–1^ in distilled water without further treatment.

### Alkali Treatment

Alkali treatments were performed using 4 and 2 wt % NaOH solutions to alter the chemical composition of the ginger fibers. Treatments were performed at a liquor/dry matter ratio of 80:1 and 80 °C for 2 h under magnetic stirring. Sequentially, alkali-treated ginger fibers were washed with distilled water until a neutral pH was reached.

### Bleaching Procedure

Ginger fibers, with and without alkali treatment, were bleached with NaClO_2_ (2.5 wt %) in an acetic buffer (pH 4.5) at 80 °C for 2 h. After bleaching, the materials were washed with distilled water until a neutral pH was reached. Sodium chlorite is the primary oxidant in the TEMPO/NaClO/NaClO_2_ system, and bleaching treatments were performed to obtain samples that could be used for estimating the chemical composition of the ginger nanofibers, both with and without alkali treatment.

### Chemical Composition

The chemical composition of the raw material, after bleaching of ginger fibers with and without alkali treatment, was determined in accordance with the standard testing recommendations of the Technical Association of Pulp and Paper Industry (TAPPI). The extractive content was determined by Soxhlet extraction with an acetone–alcohol ratio of 2:1 for 5 h following the methodology of T 204 cm-97.^40^ Delignification of all materials was performed according to established protocols^41^ in which three additions of NaClO_2_ (1 g g_dry matter_^–1^) and acetic acid (0.2 mL g_dry matter_^–1^) were performed in intervals of 1 h. The reactions were carried out at a liquor/dry matter ratio of 40:1 and 70 °C. After delignification, the materials were washed with distilled water until a neutral pH was reached. Holocellulose and α-cellulose contents were determined according to the TAPPI standard T 203 cm-99.^42^ The hemicellulose content was calculated as the subtraction of α-cellulose from the holocellulose percentage. Klason lignin was determined in a sulfuric acid solution (72 wt %) following the TAPPI standard T 222 om-02.^43^ The presented component values are based on 10 measurements for each sample, and tests were performed in triplicate.

### Yield Determination

The treatment yields (alkali treatment, bleaching, and TEMPO oxidation) were calculated according to the following equation
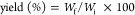
1where *W*_f_ indicates the dry weight of the sample after the alkali treatment, bleaching, and TEMPO oxidation and *W*_i_ indicates the initial dry weight of the ginger fibers.

### Optical Microscopy (OM)

Characterizations of the ginger fibers before and after the alkali treatment and TEMPO oxidation were performed using an optical microscope, Nikon Eclipse LV100N POL (Kanagawa, Japan), and the imaging software NIS-Elements D 4.30.

### Ginger Nanofiber (GNF) Production

Ginger nanofibers were obtained from the TEMPO/NaClO/NaClO_2_ system following a method described by Saito et al. with modifications and using either ginger fibers before or after alkali treatment.^44^ First, NaClO_2_ (5.0 g g_dry matter_^–1^) and TEMPO (17.5 mg g_dry matter_^–1^) were dissolved in the ginger fiber suspension at a liquor/dry matter ratio of 100:1 in the presence of a sodium phosphate buffer (0.05 M, pH 6.8). The flask was sealed and submerged in an oil bath, after which NaClO (1 mL g_dry matter_^–1^) was added and kept at a temperature of 60 °C for 72 h. After cooling the suspension to room temperature, the material was washed with distilled water until reaching a neutral pH. After the TEMPO oxidation, the GNF suspension was diluted to 0.2 wt %, homogenized with a high shear fluid homogenizer APV 2000 (SPXFlow Inc, Delavan) at 1000 bar, and collected after 1 pass. Different GNF suspensions were prepared from ginger without alkali treatment after 2 and 4 wt % alkali treatment, and they are denoted as T-GNF (TEMPO-treated GNF), AT 2%-GNF, and AT 4%-GNF (alkali and TEMPO-treated GNF with 2 or 4% NaOH), respectively. The surface charge of the ginger nanofibers was measured using a Zetasizer Nano Z (Malvern Pananalytical Ltd, Malvern, U.K.) system at room temperature (25 °C) in triplicate. Prior to the measurements, the samples were diluted to 0.05% (w/v) with distilled water.

### Preparation of Ginger Nanofiber-Based Hydrogels

Hydrogels with different grammages (40, 80, and 120 g m^–2^) were prepared by vacuum-assisted filtration of ginger cellulose nanofibers on a filter paper (Whatman grade 54, 90 mm diameter) and metal wire sieve. First, GNF suspensions were diluted to 0.1 wt % in water and dispersed under magnetic stirring for 10 min. The suspensions were then degassed and poured into a Büchner funnel with a filter paper and metal wire sieve. The suspension was filtered at room temperature (∼20 °C) for 8–24 h depending on the grammage of the hydrogel. After the GNF network was formed, the hydrogels were put into water and peeled from the filter paper. Subsequently, the hydrogels were dried at room temperature (∼20 °C), and their swelling degree was adjusted according to the characterization condition tests. A reference hydrogel was prepared from wood-based nanofibers, prepared using TEMPO oxidation and the homogenization treatment described for ginger. The reference hydrogel was prepared at 40 g m^–2^ following an equivalent procedure and used for comparison of the swelling behavior. To enhance the antimicrobial properties of the dressing material, the hydrogels prepared with T-GNF and AT 4%-GNF were functionalized with ginger essential oil (GEO). Initially, the GNF suspensions were diluted to a concentration of 0.3 wt % in water and dispersed under magnetic stirring for 10 min. The solution was then placed in a thermostatic oil bath at 40 °C, and GEO (10 wt %) was added under constant stirring for 20 min. Then, the mixed solution was sonicated using an ultrasonic processor UP400s (Hielscher Ultrasonics GmbH, Teltow, Germany) for 2 min. The hydrogel was prepared by a solution casting method and the mixed solution was cast on an acrylic plate (55 mm diameter). The hydrogel was subsequently dried at 50 °C for 12 h. For comparison, control hydrogels were also prepared by solution casting following the same procedure but without adding the ginger essential oil.

### Scanning Electron Microscopy (SEM)

The morphology of ginger fiber raw material and the cross section of the GNF hydrogel were examined using a scanning electron microscope Jeol JSM 6460LV (JEOL Ltd, Tokyo, Japan) at an acceleration voltage of 15 kV. The samples were freeze-dried and coated with platinum using an EM ACE200 sputtering instrument (Leica, Wetzlar, Germany) prior to observation to avoid electron charging. The coating was performed in a vacuum of 6 × 10^–5^ mbar under a current of 100 mA, resulting in a coating thickness of 15 nm.

### Atomic Force Microscopy (AFM)

The GNF morphology and dimensions were further analyzed using a Veeco MultiMode Scanning Probe atomic force microscope (Bruker, Santa Barbara, CA) in tapping mode with a tip model TESPA (antimony (n)-doped silicon) (Bruker, Camarillo, CA). A drop of the diluted suspension (0.01 wt %) was placed on a clean mica surface and left to dry at 22–23 °C. The nanofiber width was determined from the height images using the Nanoscope V software, and the average values and deviations presented here are based on 100 different measurements.

### Liquid Absorption Measurement

GNF hydrogels at dry state were immersed in distilled water, PBS, and BSA (50 g L^–1^) solutions at room temperature to study the liquid absorption over time. The wet weight of the hydrogels was recorded at regular intervals over 72 h. Excess liquid was removed before weighing by gently tapping the samples on a dry tissue paper. Liquid absorption was calculated as follows
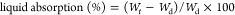
2where *W*_d_ denotes the initial weight of the dried sample and *W_t_* denotes the weight at time *t* after immersing samples in distilled water and PBS.

### Hydrogel Integrity Measurement

Hydrogel structural integrity was evaluated by measuring its weight in BSA and PBS solutions at room temperature over time. Hydrogels were incubated either in BSA or PBS solution, and the wet weight at the equilibrium state was taken as 100%. The wet weight of hydrogels was measured at regular intervals, and the percentage of weight remaining was determined by the following equation
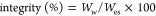
3where *W*_es_ denotes the initial weight at the equilibrium state of the sample and *W*_w_ denotes the wet weight of the sample.

### Water Retention Capacity

Hydrogel samples with a grammage of 40 g m^–2^ were allowed to swell in distilled water until they reached equilibrium. Excess water was wiped off, and then the hydrogels were left at ambient temperature for 24 h and weighed at regular intervals. The water retention capacity was calculated by the following equation
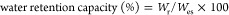
4where *W*_es_ denotes the initial weight at the equilibrium state of the sample and *W*_r_ denotes the weight retained after the sample was left to dry.

### Mechanical Testing

Compressive properties of hydrogels were analyzed using a Q800 dynamic mechanical analyzer (TA Instruments, New Castle) with the compression configuration. Samples with dimensions of 5 × 5 mm^2^ and thicknesses ranging between 2 and 5 mm were tested in wet conditions (800% of swelling degree). Tests were carried out with a 1 mN preload and a strain rate of 5% min^–1^. The compressive modulus was calculated from the slope of the initial linear region of the stress–strain curves (strain value lower than 5%). Ten measurements were taken for each sample and averaged.

Tensile properties of the hydrogels were measured using a Shimadzu Autograph AG-X universal testing machine (Shimadzu Corp., Kyoto, Japan) equipped with a load cell of 1 kN. Tests were performed at a strain rate of 2 mm min^–1^ and with a gauge length of 20 mm. The samples were in the form of strips of 6 mm width and 80 mm length and were tested in wet conditions (800% of swelling degree for comparison under similar conditions). Ten measurements were taken for each sample and averaged. Statistical analysis at a 5% significance level based on Levene’s test was used to assess the equality of variances, and the ANOVA test was performed to compare the averages of the mechanical properties.

### Antibacterial Activity Measurement

*Staphylococcus aureus* and *Escherichia coli* were streaked onto Luria Bertani (LB) agar plates and incubated at 37 °C overnight. Single bacterial colonies were picked and inoculated in 5 mL of LB broth and cultivated overnight on a shaker (300 rpm) at 37 °C. The bacterial concentration was determined by viable count, which was adjusted to correspond to 10^9^ CFU mL^–1^. The antimicrobial activity of ginger nanofibers was studied by spreading *S. aureus* and *E. coli* (10^7^ CFU in 100 μL) on Mueller Hinton Agar and placing the hydrogels onto the bacterial lawn. The plates were incubated at 37 °C for 20 h and the zones of inhibition were visualized by acquiring images at a magnification of 12.5× using an Olympus SZX9 stereo zoom microscope, (Olympus, Solna, Sweden).

Direct antimicrobial activity of the ginger material (contact-dependent) was determined on Mueller Hinton Agar following addition of *S. aureus* and *E. coli* (10^5^ CFU in 10 μL) on top of the membranes. The plates were incubated at 37 °C for 20 h, followed by image acquisition. The hydrogels were placed upside down on a new Mueller Hinton plate for 10 s and removed to determine bacterial viability. After 20 h of incubation, the bacterial growth was visualized by capturing images using an Olympus SZX9 microscope at 12.5× magnification (Olympus, Solna, Sweden).

### Primary Cell Isolation and Cultures

Primary skin cells were isolated from human tissue obtained from healthy patients undergoing routine reduction abdominoplasty at the University Hospital in Linköping, Sweden. All human tissue and cells were handled in accordance with guidelines postulated by Linköping University under approval from the Swedish Ethical Review Authority (no. 2018/97-31). Briefly, human keratinocytes and fibroblasts were isolated by mechanical and enzymatic dissection of viable epidermis and dermis under sterile conditions. For isolation of fibroblasts, skin samples were repeatedly washed in sterile PBS and then subcutaneous fat was mechanically removed. The remaining dermis was dissected into approximately two hundred 1 × 3 mm^2^ pieces, carefully avoiding epidermis and irregular structures such as large vessels, and placed in Dulbecco’s modified Eagle’s medium (DMEM, Gibco Thermo Fisher Scientific, Paisley, U.K.) with 165 U mL^–1^ collagenase (Gibco Thermo Fisher Scientific, Paisley, U.K.) and 2.5 mg mL^–1^ dispase (Gibco Thermo Fisher Scientific, Paisley, U.K.) and incubated at 37 °C, 5% CO_2_, and 95% humidity for 12 h. After enzymatic digestion, the tissue is mostly dissolved and so the suspension is centrifuged for 5 min at 200*g* and the resulting cell pellet resuspended in fibroblast medium (DMEM containing 10% fetal calf serum, 50 U mL^–1^ penicillin, and 50 mg mL^–1^ streptomycin). This was repeated twice with a fresh medium to wash the cells. The final cell pellet was dissociated, and the cells were seeded into 75 cm^2^ culture flasks (Falcon, Corning Inc; Corning, NY) in fibroblast medium and kept in an incubator at 37 °C, 5% CO_2_, and 95% humidity. The medium was changed three times per week.

For isolation of keratinocytes, samples were processed according to a modification of the protocol described by Rheinwald and Green.^45^ Briefly, samples were repeatedly washed in sterile PBS, and subcutaneous fat was mechanically removed. The remaining tissue was cut into 5 × 5 mm^2^ pieces and placed in DMEM with 2.5 mg mL^–1^ dispase at 7 °C overnight. The epidermis was then manually removed from the dermis and placed in DMEM containing 0.02% versene/0.1% trypsin (Gibco Thermo Fisher Scientific, Paisley, U.K.) and incubated for 15 min at 37 °C, 5% CO_2_, and 95% humidity. Pooled supernatants were centrifuged for 5 min at 365*g* and the resulting cell pellet washed in DMEM (Gibco Thermo Fisher Scientific). Cells were seeded into 75 cm^2^ culture flasks (Falcon, Corning Inc) with keratinocyte serum-free medium (KSFM; containing 1 mg L^–1^ epidermal growth factor, 25 mg L^–1^ bovine pituitary extract, 50 U/mL penicillin, and 50 mg/mL streptomycin; Gibco Thermo Fisher Scientific) and kept in an incubator at 37 °C, 5% CO_2_, and 95% humidity. The medium was changed three times per week.

Initial tests where cells were seeded on hydrogels were performed. Briefly, 200,000 keratinocytes or fibroblasts were seeded on 1 × 1 cm^2^ hydrogels. Following 48 h of incubation at 37 °C, 5% CO_2_, and 95% humidity, materials were fixed in 4% paraformaldehyde, dehydrated using an ethanol series (70, 95, and 99.5%), and embedded in paraffin. Then, 7 μm thick sections were mounted on microscopy slides and stained using AlexaFluor546-conjugated Phalloidin (Thermo Fisher Scientific) and 4′,6-diamidino-2-phenylindole (Thermo Fisher Scientific) to visualize cells. Samples were examined using a BX41 light/fluorescence microscope with a DP70 CCD camera (Olympus, Stockholm, Sweden), and cell nuclei were manually counted.

### Cell Viability and Proliferation

Following establishment of primary cultures, cells were enzymatically detached using 0.02% versene/0.1% trypsin and seeded in six-well plates (Falcon, Corning Inc). Cells were allowed to adhere for 24 h and were subsequently covered with Ø 10 mm discs of T-GNF or AT 4%-GNF and cultured for 72 h. Keratinocyte and fibroblast cultures in three replicate wells without addition of material served as controls. Every 24 h, for 72 h in total, the cells were detached using a 0.02% versene/0.1% trypsin solution at 37 °C for approximately 10 min, stained with trypan blue to distinguish viable cells, and counted using an EVE automated cell counter (NanoEnTek, Waltham, MA). All experiments were performed in biological triplicate (separate cell cultures) and methodological duplicate (staining and cell counting). Numbers of viable cells were recorded and analyzed using Prism 8.4.2 (Graphpad LLC, LaJolla, CA). A two-way ANOVA coupled with a Holm-Sidak post-test was used to compare cell numbers over time in all groups of the same cell type, and *p* < 0.05 was considered significant. Cell numbers in the treatment groups were normalized to nontreated controls and expressed as a proliferative index according to the following equation
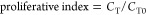
5where *C*_T_ denotes the mean number of viable cells at the analyzed time point and *C*_T0_ denotes the mean number of viable cells at the starting time point. All values are plotted as mean ± standard deviation (SD) unless otherwise stated.

### Cell Migration Assay

To assess the effects of T-GNF or AT 4%-GNF on cell migration, fibroblasts and keratinocytes were seeded in separate triplicate six-well plates as described above. Cells were cultured until confluency, and a denuded scratch was produced using a p200 pipette tip across the center of the wells. Cultures were covered with the hydrogels and examined at 0, 24, 48, and 72 h using an IX51 light microscope with a DP70 CCD camera (Olympus, Stockholm, Sweden). Photos were captured at 10× magnification and analyzed using ImageJ 1.52p (NIH) to measure the remaining cell-free area in the denuded streak. The areas were normalized to the area at time 0 according to the following equation
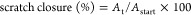
6where *A*_t_ denotes the mean remaining denuded area at the experimental time and *A*_Start_ denotes the initial mean wound area. A mixed-effect restricted maximum likelihood (REML) model coupled with a Holm-Sidak post-test was used to compare cell numbers over time in all groups of the same cell type, and *p* < 0.05 was considered significant. All values are plotted as mean ± standard deviation (SD) unless otherwise stated.

## Results and Discussion

### Production and Characterization of Ginger Nanofibers

**Figure 1:**
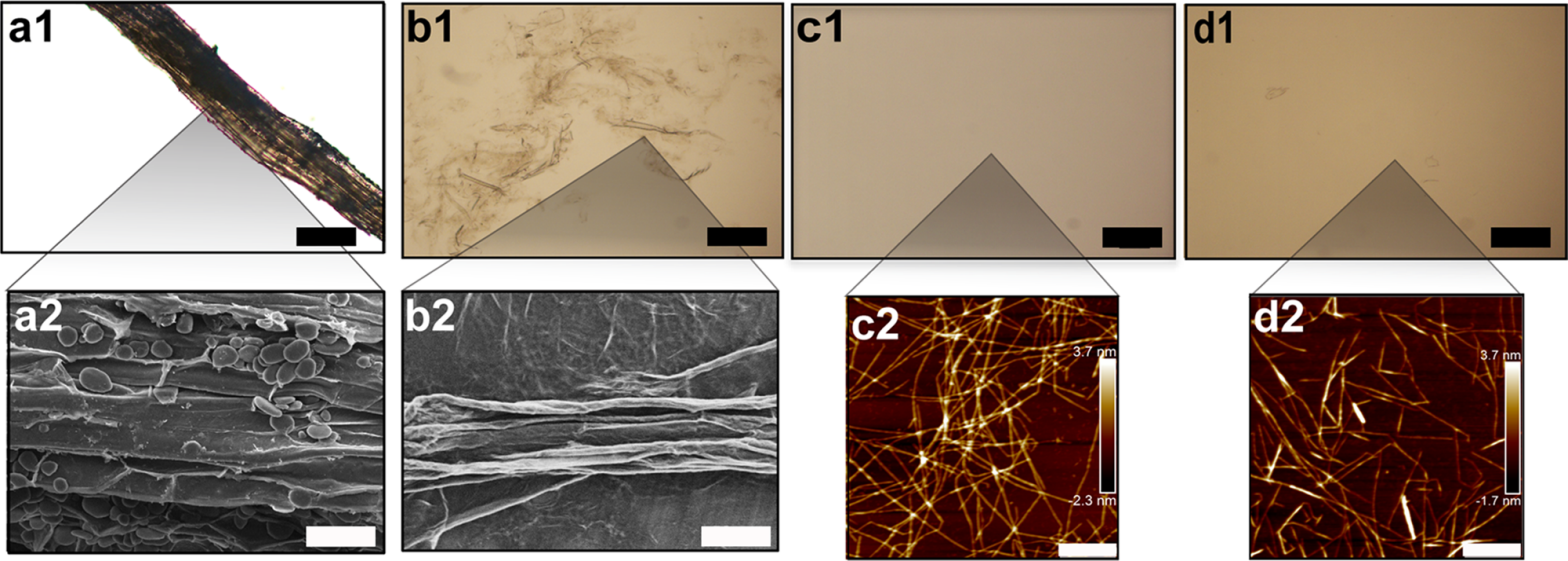
OMs of the ginger raw material (a1) before and (b1) after the alkali treatment step. OM images of the homogenized nanofiber suspension after (c1) only TEMPO treatment (T-GNF) and (d1) alkali and TEMPO treatment (AT 4%-GNF) (scale bar: 200 μm). SEM images of dried ginger fibers: (a2) nonalkali treated and (b2) after alkali treatment (scale bar: 50 μm). AFM images of dried nanofiber suspensions (c2) T-GNF and (d2) AT 4%-GNF (scale bar: 400 nm).

Hydrogels with different liquid absorption capacities are interesting for wound dressing applications. Different types of wounds and different stages of the healing process may have different requirements for an optimal dressing. The process for material preparation can result in different material properties, and natural starting materials can have different advantageous properties. We sought to leverage this knowledge to develop a multifunctional material aimed at advanced wound dressings, sourced and produced with sustainability in mind. Ginger nanofibers were produced by TEMPO oxidation combined with high-pressure homogenization before and after alkali treatment. The effects of the different processing routes and the nanofiber compositional varieties derived from the alkali treatments were further studied with regard to hydrogel properties (Figure 1). The chemical composition without alkali treatment was 25.1 ± 0.5% α-cellulose, 40.5 ± 0.6% hemicellulose, 5.5 ± 0.9% Klason lignin, and 5.1 ± 1.3% extractives. These results are in agreement with those of Abral et al., who found cellulose and hemicellulose contents to be 30 and 44%, respectively.^35^ However, Zaki, Abdullah, and Ahmad reported a higher value of cellulose (65.2%) and a lower hemicellulose value (10.1%).^46^ The differences in proportions of each individual component can be explained by a number of factors related to the analysis methodology or the sample itself. These factors include country of origin, harvesting conditions, industrial processing, and whether the ginger is fresh, dried, or processed.^47^ Besides the high contents of cellulose and hemicellulose, ginger has a significant amount of starch varying between 30 and 60%.^48,49^ It is generally accepted that alkali treatment changes the composition of the fibers by targeting the noncellulosic components, such as starch, hemicellulose, and pectin.^50^ Alkali treatment was applied to ginger fibers before TEMPO oxidation, aiming to reduce the hemicellulose content and alter the characteristics of the nanofibers. This directly influenced the properties of the hydrogels prepared with them. The content of hemicellulose after oxidation was 11.0 ± 0.8%, which supports that the alkali treatment reduces noncellulosic components. The process yields, with and without alkali-treated ginger as feedstock, are presented in Table 1.

**Table 1:** Total Yield and Weight after Each Step of the Ginger Nanofiber Production Process

samples	initial weight [g]	weight after alkali treatment [g]	weight after TEMPO oxidation [g]	total yield [%]
T-GNF	10		4.3	43.3
AT 4%-GNF	10	1.5	0.6	6.4

The lower yield (6.4%) obtained using alkali-treated ginger is attributed to the material loss during the alkali step. However, this value is higher than the ginger nanofiber yield of 3.1% reported by Jacob et al. who also applied an alkali treatment on ginger fibers under similar conditions to reduce the noncellulosic components.^32^

The optical micrographs (OMs) are presented in Figure 1, showing the raw material, the ginger fiber with and without alkali treatment, and GNFs, together with atomic force microscopy (AFM) height images (Figure 1c2,d2).

From Figure 1c1, it is possible to note that alkali treatment generated microsized structures composed of fragments of parenchyma cells and fibers of different sizes. The representative SEM images before and after alkali treatment are shown in Figure 1a2 and b2, respectively. Starch granules can be observed with an oval shape and a smooth surface without fissures, typically associated with fibers in ginger.^49,51^ A comparison between Figure 1a2 and b2 indicates that besides reducing the hemicellulose content, the alkali treatment also reduced the starch content since the starch granules are absent in the alkali-treated fibers. Furthermore, the OMs of the GNF suspensions obtained from TEMPO oxidation directly followed by homogenization and after alkali treatment of ginger are presented in Figure 1c1 and d1, respectively. In both conditions, it was not possible to observe any large structures after the TEMPO treatment confirming that their size was reduced to the nanoscale.

The overview AFM images of GNFs together with their size distribution (Figure S1) are provided in the Supporting Information. The size of the GNFs was measured from AFM height images (Figure 1c2,d2) and the averaged width values of T-GNFs and AT 4%-GNFs were 1.7 ± 0.7 and 2.0 ± 1.1 nm, respectively. Overall, it can be noted that the widths were comparable for both samples (Figure S1, Supporting Information). Additionally, the ginger nanofiber width is in agreement with the values of TEMPO nanofibers isolated from wood reported elsewhere.^44,52^

### Liquid Absorption Capacity of Ginger Nanofiber Hydrogels

One of the most important properties of hydrogels for wound dressing applications is the water/liquid absorption, sometimes expressed as the swelling degree, which directly reflects the hydration ability.^53^ A moist environment allows for cell migration of epithelial cells and leukocytes, and distribution of growth factors, into the wound bed, thus facilitating the wound healing process.^54^

**Figure 2:**
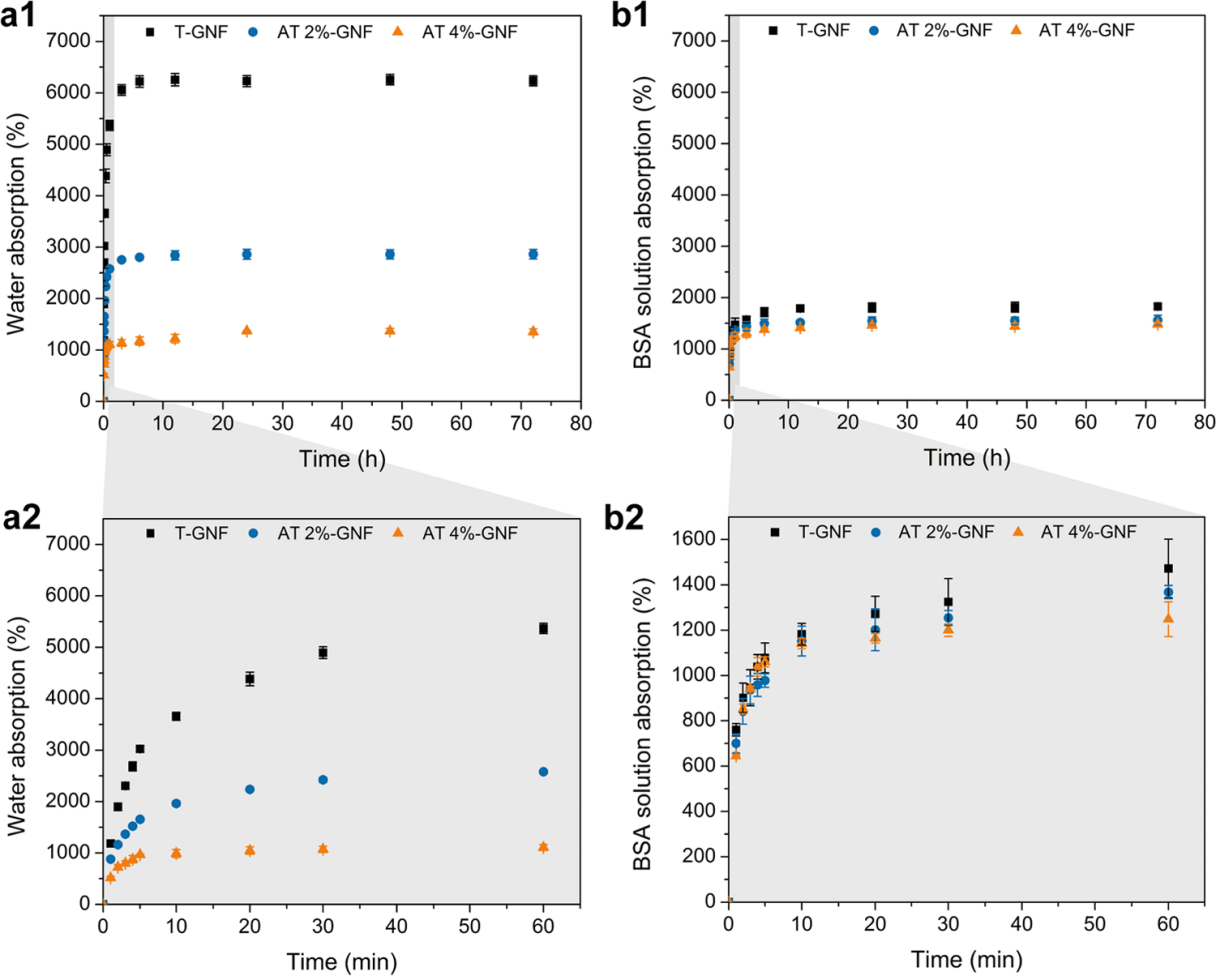
Liquid absorption of the hydrogels (40 g m^–2^) in (a1) water and (b1) BSA solution. Liquid absorption of hydrogel curves expanded for the first hour in (a2) water and (b2) BSA solution.

The liquid absorption over time of hydrogels prepared from GNFs isolated directly after TEMPO treatment (T-GNF) and alkali treatment using 2 wt % (AT 2%-GNF) and 4 wt % (AT 4%-GNF) sodium hydroxide is shown in Figure 2.

The highest water absorption capacity was obtained by the T-GNF hydrogel (∼6200%), which was about five times higher than the value obtained by the AT 4%-GNF hydrogel (∼1350%), as well as that measured for the reference hydrogel prepared from wood-based nanofibers (∼1250%) (Figure 2a1). From Figure 2a2, it is evident that the T-GNF hydrogel reached the same absorption capacity value as that of AT 4%-GNF at equilibrium in the first few minutes after immersion in water. It is worth highlighting this because the T-GNF hydrogel showed water absorption capacity of 62 times its dry weight, owing to which T-GNF can be compared to a superabsorbent hydrogel.^55−57^ This is an interesting result as the hydrogel was prepared only from natural polymer without using any cross-linker, swelling agent, or highly hydrophilic synthetic polymer and through a simple methodology. The high water absorption of T-GNF is likely associated with the natural composition of ginger that is rich in both hemicellulose and starch in contrast with AT 4%-GNF and the wood-based reference material, where these components are reduced (and in the case of wood, the starch content is absent). Zhang et al. prepared a superabsorbent hydrogel based on the high swelling capacity of hemicellulose.^58^ Hydrophilic polymers, such as starch, carrageenan, and poly-acrylamide, are commonly added to hydrogels to enhance their swelling capacity.^59^ These results suggest that the natural composition of ginger can be beneficial for the preparation of hydrogels with a high water absorption capacity. Additionally, alkali treatments can be used to effectively alter the liquid absorption capacity of the hydrogels by modifying the chemical composition of the raw materials.

The swelling behavior was also evaluated in BSA solution to more closely resemble the wound environment in terms of a higher abundance of proteins (Figure 2b1,b2). The difference between the hydrogel liquid absorption capacities was less pronounced in BSA solution, and the same behavior was also observed for PBS solution (Figure S2, Supporting Information). However, the T-GNF hydrogel still showed the highest swelling degree at equilibrium when compared with AT 2%-GNF and AT 4%-GNF hydrogels. The swelling at equilibrium was overall lower in BSA than in water, which could be attributed to the repulsive electrostatic interactions between the BSA molecules and the hydrogels. The swelling behavior of neutral hydrogels is controlled by opposing forces resulting from the spontaneous mixing of the fluid molecules with the polymer chains and the retractive forces developed inside the structure. The equilibrium swelling state is reached when these forces are balanced. The presence of ionic groups in the hydrogel structure and ions in the surrounding media generate additional forces that affect the balance, thus influencing the liquid absorption properties.^60^ Both hydrogels are negatively charged as indicated by the negative ζ-potentials: −44.8 ± 0.7 mV for T-GNF and −65.6 ± 0.7 mV for AT 4%-GNF. All proteins contain positively and negatively charged groups, and their effective charge is a result of the balance of these groups at a given pH. The BSA solution was prepared in water, and the effective charge value at pH higher than the isoelectric point of BSA (4.7)^61^ has been reported to be around −7.0 mV.^62^ Thus, the ionic interactions between the carboxyl groups on the GNF surface with charged groups in the BSA molecules might have counteracted the swelling of the hydrogel structure, resulting in decreased liquid absorption. Similarly, Kono et al. reported a reduction of the swelling degree due to the repulsive electrostatic interactions between the BSA molecules and the hydrogel that had carboxylate anions as the predominant charged species.^63^

The structural integrity of the hydrogels was evaluated as the weight remaining after immersing the samples either in BSA or in PBS solution (Tables S1 and S2, Supporting Information). Within 40 days, the wet weight remained constant for all of the hydrogels, indicating no material loss in either incubation condition. It is noteworthy that the hydrogels maintained their structural integrity for long periods of time. The water retention capacity reflects the ability of a dressing material to hold water molecules within its structure, which may be important for keeping the wound moist. The water retention capacities of T-GNF (16 ± 3.6%) and AT 4%-GNF (12 ± 3.3%) hydrogels were comparable, after exposure to air, at room temperature for 24 h (Figure S3, Supporting Information).

**Figure 3:**
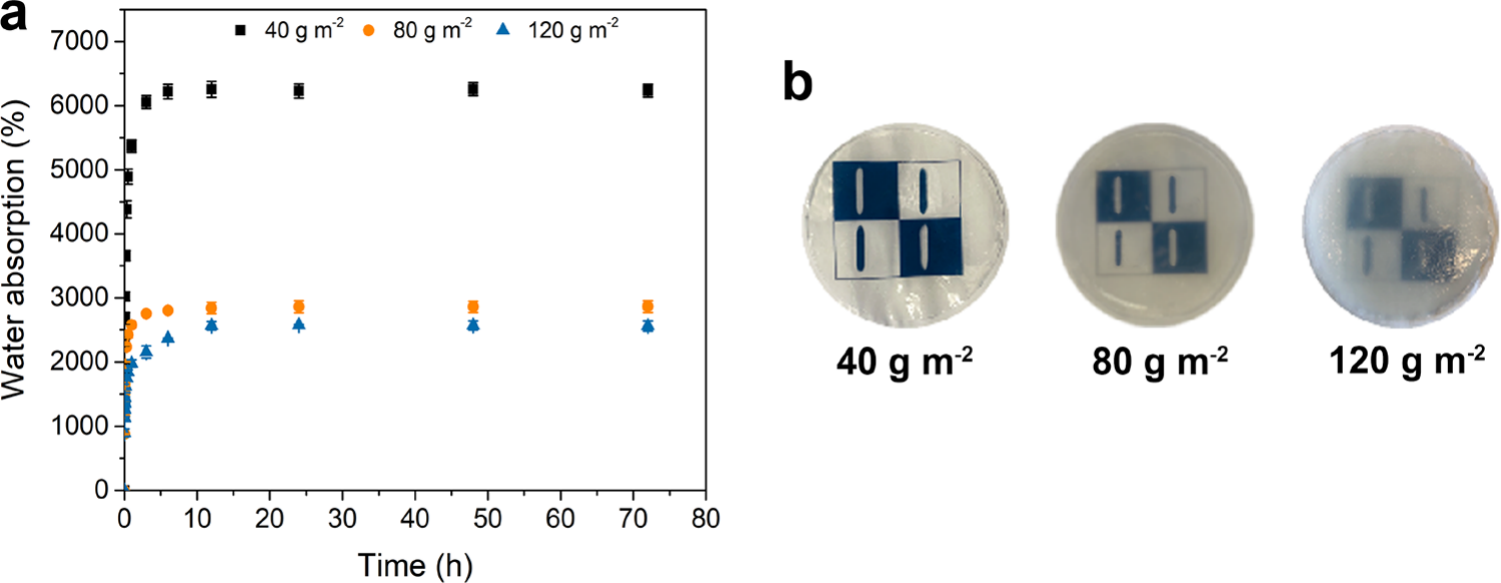
(a) Water absorption as a function of time for T-GNF hydrogels at different grammages. (b) Photographs of the T-GNF hydrogels at different grammages after the equilibrium swelling degree was reached.

Since the T-GNF hydrogel showed a higher liquid absorption capacity, the influence of the hydrogel grammage on water absorption was investigated and is presented in Figure 3.

From Figure 3, it is noted that the highest swelling degree was reached by the hydrogel with the lowest grammage (40 g m^–2^). The increase in the grammage led to a significant decrease in swelling. This behavior can be due to a higher degree of nanofibers, which favors the formation of connected points, and, in turn, increases the rigidity of the network and reduces the swelling capacity. It should be noted that controlling the hydrogel grammage could be another approach to adjust the liquid absorption capacity. Comparing the hydrogels with different grammages (Figure 3b), it is possible to observe that the transparency reduced as the grammage increased. Thus, besides the highest swelling degree, the hydrogel produced with T-GNF at 40 g m^–2^ presented good transparency, which is advantageous for dressing materials as it allows for monitoring of the wound during the healing process (Figure S4, Supporting Information).

**Figure 4:**
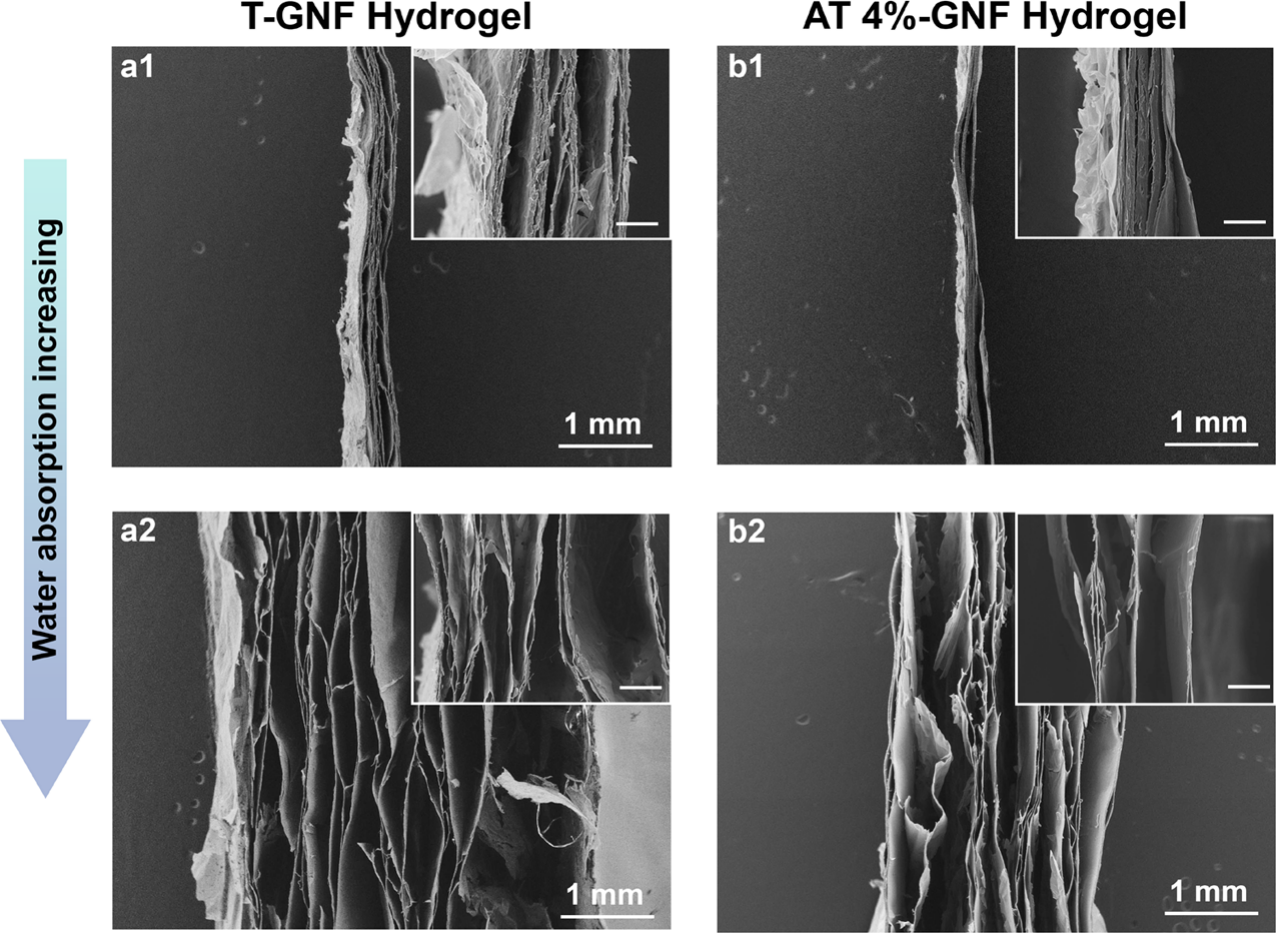
SEM images of ginger nanofiber hydrogels of 120 g m^–2^ grammage showing the swelling mechanism due to water absorption. (a1) T-GNF and (b1) AT 4%-GNF at 800% of swelling degree and (a2) T-GNF and (b2) AT 4%-GNF at equilibrium swelling degree. Scale bar of the insets: 100 μm.

The morphology of the hydrogels produced with T-GNFs and AT 4%-GNFs at a low swelling degree (800%) and after they reached the equilibrium swelling degree in water was investigated using SEM and is presented in Figure 4.

The cross sections of both GNF hydrogels displayed a layer-like structure after vacuum-assisted filtration assembly (Figure 4). The layered networks are formed as the water evaporates via fiber–fiber bonding due to secondary attraction forces, including hydrogen bonds, that develop between the nanofibers.^64^ This layered nanofiber network structure has been documented by other studies in the literature.^65−69^ Comparing the different conditions shown in Figure 4, it is possible to observe the swelling mechanism in which the layers are more spaced out with increasing water absorption. Additionally, it can be noted that the T-GNF hydrogel (Figure 4a2) is thicker than the AT 4%-GNF hydrogel (Figure 4b2) at the same swelling degree, which corroborates the higher liquid absorption capacity of the former.

### Mechanical Properties

**Figure 5:**
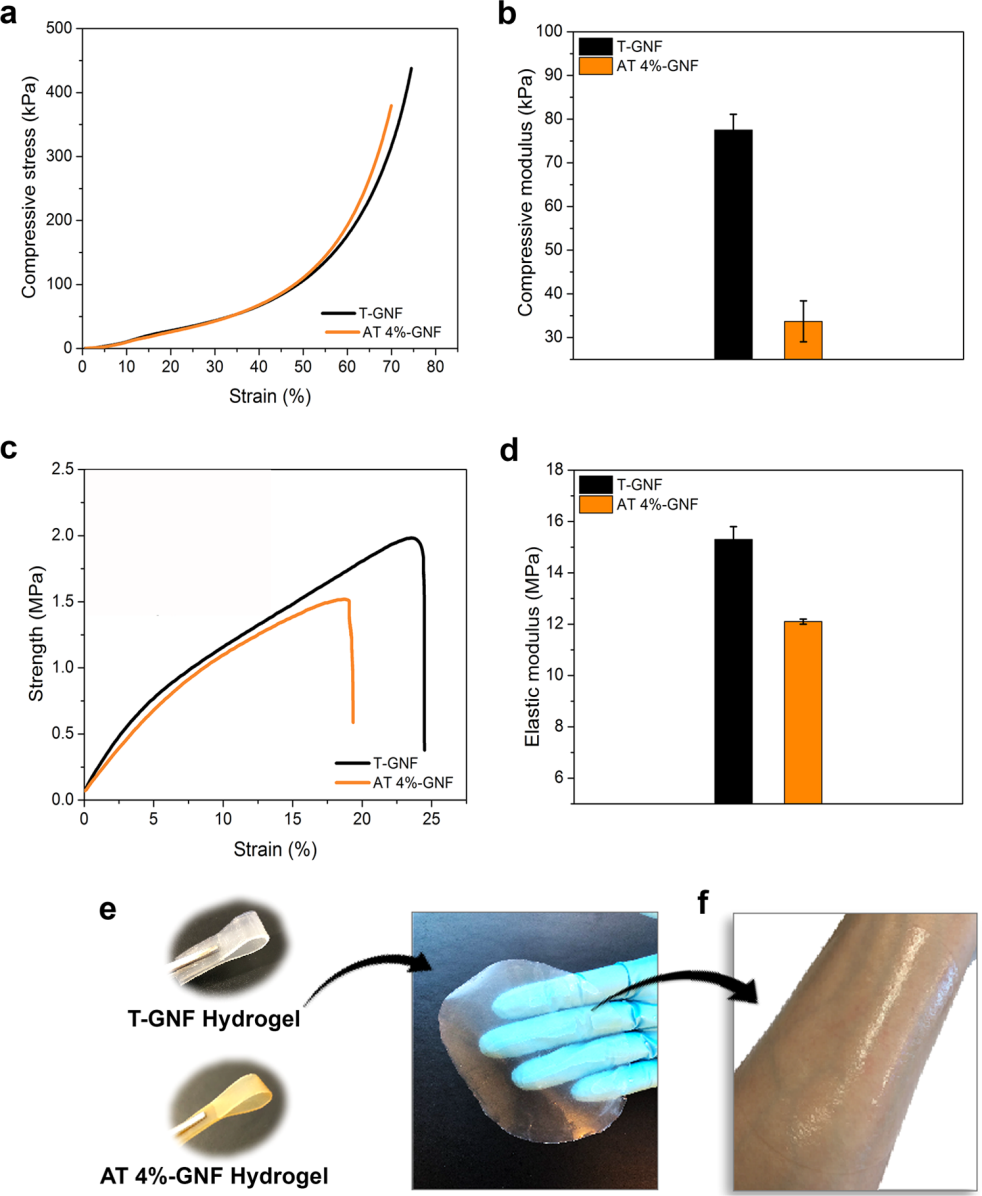
Mechanical properties and hydrogel appearance. (a) Representative compressive stress–strain curves and (b) the compressive modulus of the GNF hydrogels at 40 g m^–2^ determined in wet conditions. (c) Representative tensile stress–strain curves and (d) tensile properties of the GNF hydrogels at 40 g m^–2^ determined in wet conditions. (e) Photographs of the T-GNF and AT 4%-GNF hydrogels and (f) application of the T-GNF hydrogel on the forearm.

The mechanical properties of the hydrogels and the representative tensile and compressive stress–strain curves are displayed in Figure 5 and Table S3 (Supporting Information). The initial linear region of the stress–strain curves used to calculate the compressive modulus is provided in Figure S5 (Supporting Information). From Figure 5, it can be noted that the T-GNF hydrogel exhibited better compressive properties, displaying a compressive modulus of 77.5 ± 3.6 kPa, which is more than two times the value obtained for the AT 4%-GNF hydrogel (33.7 ± 4.7 kPa). It has been demonstrated that hemicellulose facilitates the nanofibrillation of wood and contributes to enhanced stiffness and strength of composites.^70^ Even though the length of nanofibers cannot be determined from AFM images, due to entanglements, the T-GNFs (Figure 1c2) appear to have been isolated with a preserved length that allows for increased interconnectivity compared to AT 4%-GNFs (Figure 1d2), which showed shorter nanofibers. Longer nanofibers with a high aspect ratio result in more entanglements and contact points, leading to stronger networks.^66^ Tensile properties such as the strength and strain to failure are important for wound dressings as the material should not break during the fixation process, wearing, or removal.^71^

From Figure 5c,d, The T-GNF hydrogel exhibited excellent mechanical performance with a tensile strength of 2.1 ± 0.2 MPa and an elastic modulus of 15.3 ± 0.3 MPa, compared to the AT 4%-GNF hydrogel with a tensile strength of 1.6 ± 0.1 MPa and an elastic modulus of 12.1 ± 0.1 MPa. Sun et al. reported elastic modulus in the range of 0.4–3.0 MPa and tensile strength less than 0.25 MPa measured in wet conditions of the TEMPO-oxidized cellulose nanofiber films obtained by solvent casting.^72^ These values were lower than those obtained for GNFs, and the difference might be associated with the method used for cellulose nanofiber processing, which affects the properties (e.g., mechanical, optical) of the resulting materials.^73^ Studies in the literature comparing the effects of different processes, such as casting and filtration, on the mechanical properties have reported that the filtration technique leads to a mechanically stronger nanofiber network relative to the casting method.^74−76^ According to Sehaqui et al., the network properties are very sensitive to nanofiber orientation, and the higher modulus and tensile strength are likely attributed to the increased in-plane orientation of the nanofibers.^74^

Moreover, the elastic modulus of skin reported in the literature varies considerably depending on the type and conditions of the mechanical test, and for the tensile test, it ranges from 4.6 and 20 MPa.^77^ Since the values reported here are within this range, hydrogels possess the potential for wound dressing applications. Additionally, the hydrogels presented good flexibility (Figure 5e) and shape retention and were gently pliant to the skin (Figure 5f).

### Antibacterial Effects

**Figure 6:**
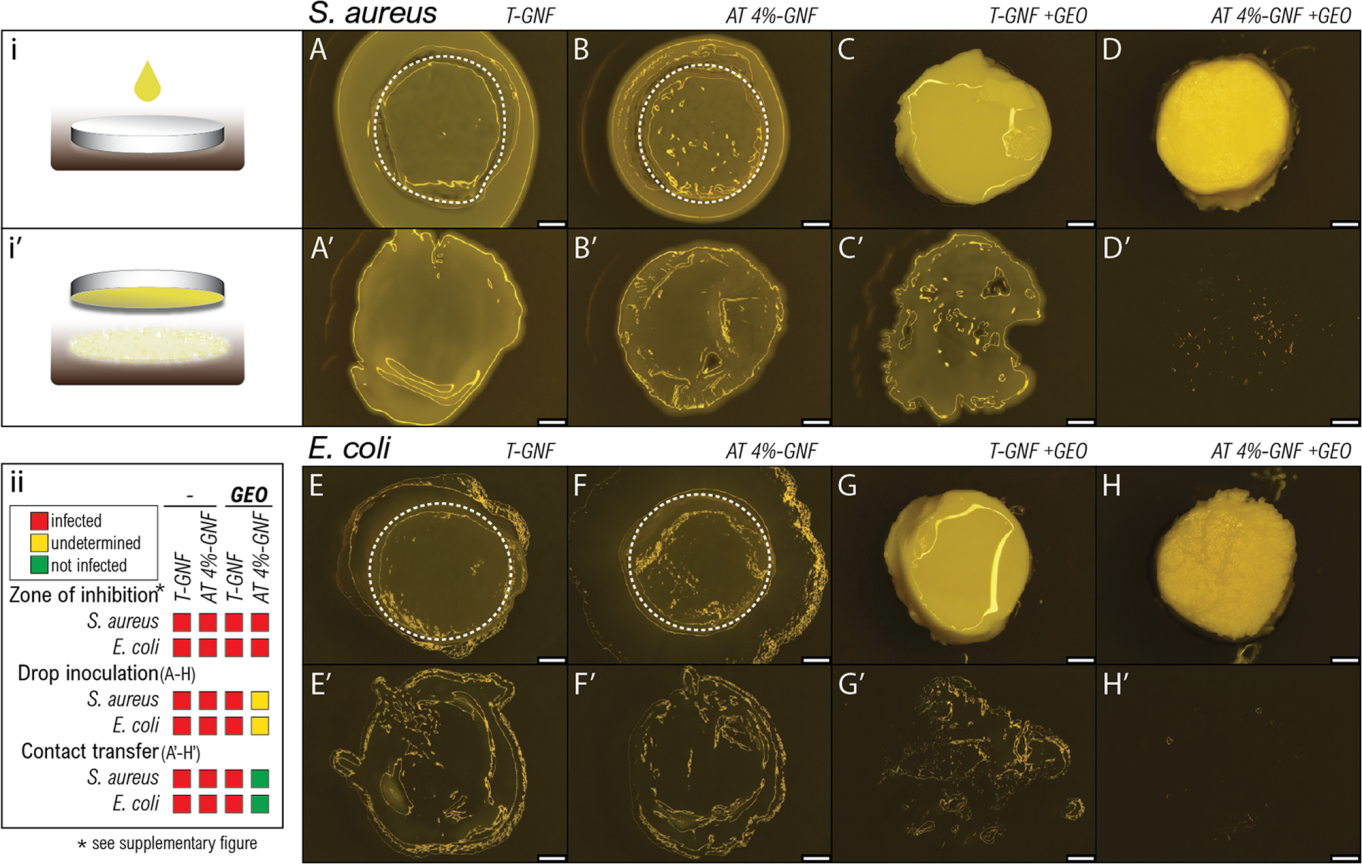
Images from microbiological assays against *S. aureus* and *E. coli* for T-GNF and AT 4%-GNF hydrogels before and after functionalization with ginger essential oil (GEO). Method sketches of (i) drop inoculation of hydrogels (upper rows: A–H) and (i′) subsequent contact-dependent transfer of microbes to fresh agar (lower rows: A′–H′). (A–D) *S. aureus*-infected hydrogels and (A′–D′) contact transfer of *S. aureus* and (E–H) *E. coli*-infected hydrogels and (E′–H′) contact transfer of *E. coli*. Panel (ii) shows the summary of antimicrobial results. * zone of inhibition assay is shown in Supporting Information S6. Dashed lines represent the approximate area of hydrogel beneath the bacterial colony; scale bars, 1 mm.

To evaluate the antibacterial activity of hydrogels, microbiological assays were performed against Gram-positive *S. aureus* and Gram-negative *E. coli* (Figure 6), and the overview images and the zone of inhibition are provided in Figure S6, Supporting Information.

Ginger essential oil (GEO) was chosen to functionalize the hydrogels since it has been reported to have antimicrobial activity.^29^ Before and after functionalization, no clear zone of inhibition could be observed for T-GNF and AT 4%-GNF hydrogels (Figure S6), indicating no measurable inhibitory activity against *S. aureus* and *E. coli*. In contrast, Abral et al. and Jacob et al. reported good antimicrobial performance of ginger nanofiber films against different bacteria species.^33,35^ There are many factors affecting the antibacterial activity of ginger, such as processing methods, the type of microorganisms,^35^ and/or raw material storage conditions.^78^ Plausible reasons for the loss of antibacterial activity of the ginger nanofiber hydrogels in this study, compared to previously reported studies, could be differences in nanofiber production methods or the use of raw material stored for longer time periods as ginger is not grown in Sweden.^31,33^ Bacterial growth could not be determined after drop inoculation of hydrogels (Figure 6A–H). However, the subsequent contact-dependent transfer of microbes to fresh agar (Figure 6A′–H′) revealed a bactericidal effect after GEO functionalization, indicated by the absence of bacterial growth for AT 4%-GNF (Figure 6D′,H′), compared to the bacterial growth pattern found in the other materials (Figure 6A′–C′,E′–G′).

Variation in the chemical composition of the hydrogels might explain their different antibacterial activity. Overall, GEO appears to contribute to antimicrobial hydrogels, hindering bacterial growth on the hydrogels, without releasing active antimicrobial substances under these conditions. Infected wounds are commonly treated with antibiotics, and the rising problem of antibiotic resistance is alarming. The use of completely biobased resources may be a safer way to obtain advanced wound dressing material with antimicrobial properties while minimizing cytotoxic side effects.^79−81^ The use of GEO for antimicrobial activity enhancement of completely ginger-based hydrogels demonstrates the potential to utilize multiple intrinsic properties of raw material to develop functional materials.

### In Vitro Cytocompatibility Study

**Figure 7:**
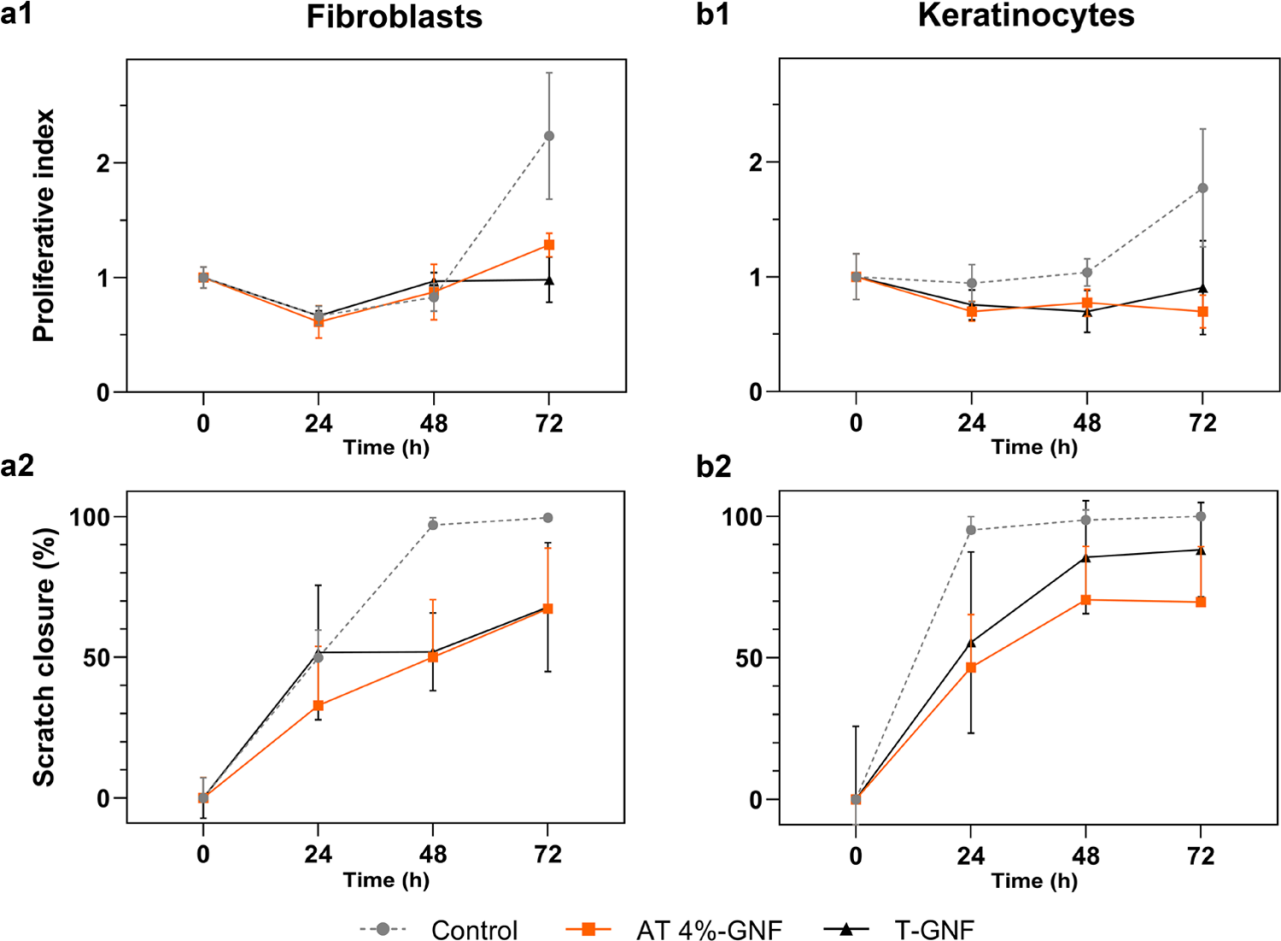
Effects of T-GNF and AT 4%-GNF hydrogels on fibroblast (a1) proliferation and (a2) migration and keratinocyte (b1) proliferation and (b2) migration. *n* = 4.

The potential use of the GNF hydrogels for wound dressing application requires an initial assessment of cytocompatibility. Since wound healing is primarily executed by the proliferation and migration of keratinocytes and fibroblasts, the migratory and proliferative behavior of primary keratinocytes and fibroblasts from human abdominal skin were separately evaluated in the presence of the T-GNF and AT 4%-GNF hydrogels by culturing them in direct contact with the two GNF hydrogels. Initial tests where cells were seeded on top of the hydrogels revealed a low degree of adhesion with no increase in cell numbers over time (Supporting Information S7). This finding indicates that the hydrogels are unsuitable for use as cell-loaded scaffold material but suggests desirable properties for dressing applications, where incorporation of tissue from the healing wound may cause difficulties in dressing changes. A proliferation and viability assay where viable cells were counted over time and a migration assay (scratch wound repopulation) were employed for the analysis of the cell cultures, and the results are presented in Figure 7.

The proliferative index of keratinocytes and fibroblasts over time is shown in Figure 7a1,b1. The interpretation of the statistical analysis revealed that both time (49%, *p* < 0.0001) and treatment (7.8%, *p* < 0.05) significantly contribute to the total variation of the proliferative index. Neither T-GNF nor AT 4%-GNF hydrogels affected fibroblast or keratinocyte proliferation before the 72 h time point. Proliferation of fibroblasts was significantly impaired after 72 h (Figure 7a1; *p* < 0.05), with a proliferative index of 0.98 ± 0.20 for T-GNF, 1.28 ± 0.10 for AT 4%-GNF, and 2.23 ± 0.55 for the control. Proliferation of keratinocytes was significantly impaired after 72 h (Figure 7b1; *p* < 0.05), with a proliferative index of 0.91 ± 0.41 for T-GNF, 0.70 ± 0.14 for AT 4%-GNF, and 1.77 ± 0.51 for the control (Figure 7a1). Migration of fibroblasts and keratinocytes over time is shown in Figure 7a2,b2. Overall, significant differences were seen for both time and treatment (*p* < 0.0001). For fibroblasts, the scratch closure of the T-GNF group was significantly lower compared to that of the control at 48 h (52 ± 14 vs 97 ± 2.6%, *p* < 0.005). The AT 4%-GNF group was significantly reduced at 48 h (50 ± 20 vs 97 ± 2.6%, *p* < 0.001) and 72 h (67 ± 22 vs 100 ± 0.9%, *p* < 0.05). For keratinocytes, the scratch closure for the T-GNF group was significantly reduced at 24 h (55 ± 32 vs 95 ± 4.8%, *p* < 0.005) and 72 h (88 ± 17 vs 100 ± 0%, *p* < 0.05). For AT 4%-GNF, the scratch closure was significantly reduced at 24 h (47 ± 19 vs 95 ± 4.8%, *p* < 0.0001), 48 h (71 ± 19 vs 99 ± 3.6%, *p* < 0.001), and 72 h (70 ± 20 vs 100 ± 0%, *p* < 0.001). The method of placing the GNF hydrogels on top of the cells can itself interfere with normal culture conditions.^82^ The added barrier created by the GNF may interfere with the oxygen diffusion rate and the turnover of soluble factors near the cells. In an in vivo situation, the skin is fed by nutrients from the vascularized underlying tissue but the in vitro model is limited by free diffusion of oxygen through, and soluble factors added into, the culture medium. This may explain diminished migration and proliferation and help explain some of the limits to scratch closure in the migration assay. In light of these results, the control group without hydrogels can be considered evidence of a viable and healthy primary cell population, but comparisons between results with and without hydrogels should be carefully employed. More interesting are any differences between the GNF hydrogels and the overall viability and migratory capacity of the cells. Overall, the GNF hydrogels did not display cytotoxicity (Figure 7; Figures S8 and S9 Supporting Information). However, proliferation and migration of keratinocytes and fibroblasts were not increased; instead, the covering of culture resulted in somewhat decreased cell numbers. Wound healing is a complex process, and further evaluation both in vitro and in vivo would be imperative in future studies to establish the suitability of GNF hydrogels as wound dressings and better understand the effects of T-GNF and AT 4%-GNF hydrogels on healing.

## Conclusions

Hydrogels based on TEMPO-oxidized ginger nanofibers were successfully prepared via vacuum-assisted filtration without any cross-linker and with advantageous properties for wound dressing applications. It was shown that the liquid absorption capacity of the hydrogels could be adjusted by altering the chemical composition of ginger fibers with alkali treatment prior to nanofiber isolation and subsequent hydrogel formation. Furthermore, the grammage of the hydrogels was shown to dictate the absorption capacity. The hydrogel produced with ginger without alkali treatment (T-GNF hydrogel) of 40 g m^–2^ grammage showed the highest water absorption of 62 times its initial weight and reached a value that was five times higher than the one obtained with the reference wood nanofiber hydrogel. A high swelling capacity was achieved by preserving the noncellulosic components such as starch and hemicellulose naturally found in ginger when preparing the nanofibers and their hydrogels. Additionally, the T-GNF hydrogels showed good mechanical properties with tensile strength of 2.1 ± 0.2 MPa and elastic modulus of 15.3 ± 0.3 MPa. The antimicrobial activity of ginger was not preserved after nanofiber separation, as observed from microbiological assays. However, functionalization using ginger essential oil improved antimicrobial performance against *S. aureus* and *E. coli*, and the absence of bacterial growth suggests that the AT 4%-GNF hydrogel was bactericidal. However, additional experiments are needed to better understand and optimize the functionalization of ginger nanofiber hydrogels using essential oil. Cytocompatibility evaluation showed that T-GNF and AT 4%-GNF hydrogels did not significantly affect fibroblast proliferation. Meanwhile, migration of keratinocytes was more beneficial when in contact with the T-GNF hydrogel than AT 4%-GNF.

The current study highlights an upscalable environmentally friendly way to prepare completely ginger-based nanofiber hydrogels that combine functions attractive for wound dressing, such as tunable absorption, flexibility, and transparency, while being nontoxic and mechanically stable in moist conditions. Moreover, potential additional functionalization could further be explored with the aim of improving wound healing.

## Supporting Information Available

AFM images and size distribution of the nanofibers obtained with and without alkali-treated ginger; PBS solution absorption of the T-GNF and AT 4%-GNF hydrogels; hydrogel integrity in BSA and PBS solutions; water retention of the T-GNF and AT 4%-GNF hydrogels; photographs of the T-GNF hydrogels at 40 g m^–2^ grammage after reaching the equilibrium state; representative compressive stress–strain curves enlarged at 30% strain; compilation of the hydrogel mechanical properties; microbiological assays for T-GNF and AT 4%-GNF hydrogels before and after functionalization with ginger essential oil (overview and inhibition zones); investigation of cell attachment to the T-GNF hydrogel; and images from scratch assay showing migration of fibroblasts and keratinocytes at different time points (PDF)
T-GNF hydrogel applied on a forearm (AVI)

## Supplementary Material

bm1c00215_si_001.pdf

bm1c00215_si_002.avi

## Abbreviations Used

TEMPO: (2,2,6,6-tetramethylpiperidine-1-oxyl radical)-mediated oxidation
BSA: bovine serum albumin
SEM: scanning electron microscopy
AFM: atomic force microscopy
PBS: phosphate-buffered saline
S. aureus: Staphylococcus aureus
E. coli: Escherichia coli
GEO: ginger essential oil
