# Supplementary material for: Multifunctional Ginger Nanofiber Hydrogels with Tunable Absorption: The Potential for Advanced Wound Dressing Applications
Source: Biomacromolecules. 2021 Jul 13;22(8):3202–15. doi: 10.1021/acs.biomac.1c00215 (PMC8382245; doi:10.1021/acs.biomac.1c00215)
Supplement: Supplementary file 1 — bm1c00215_si_001.pdf [file bm1c00215_si_001.pdf]

Multifunctional Ginger Nanofiber Hydrogels with Tunable Super Absorption: The Potential for Advanced Wound Dressing Applications

Paula Squinca [†,^,∥], *Linn Berglund* [†,*], *Kristina Hanna* [‡], *Jonathan Rakar* [‡], *Johan Junker* [‡], *Hazem Khalaf* [§], *Cristiane S. Farinas* [^,∥] *and Kristiina Oksman* [†,∇]

†Division of Materials Science, Department of Engineering Sciences and Mathematics, Luleå University of Technology, SE-971 87 Luleå, Sweden

‡Center for Disaster Medicine and Traumatology, Department of Biomedical and Clinical Sciences, Linköping University, SE-581 85 Linköping, Sweden

§Cardiovascular Research Centre, School of Medical Sciences, Örebro University, SE-703 62 Örebro, Sweden

^Embrapa Instrumentation, Rua XV de Novembro 1452, 13561-206, São Carlos, SP, Brazil

∥Graduate Program of Chemical Engineering, Federal University of São Carlos, Rod. Washington Luís-km 235, 13565-905, São Carlos, SP, Brazil

∇Department of Mechanical & Industrial Engineering, University of Toronto, 5 King's College Road, Toronto, ON M5S 3G8, Canada.

Corresponding Author

*E-mail: linn.berglund@ltu.se

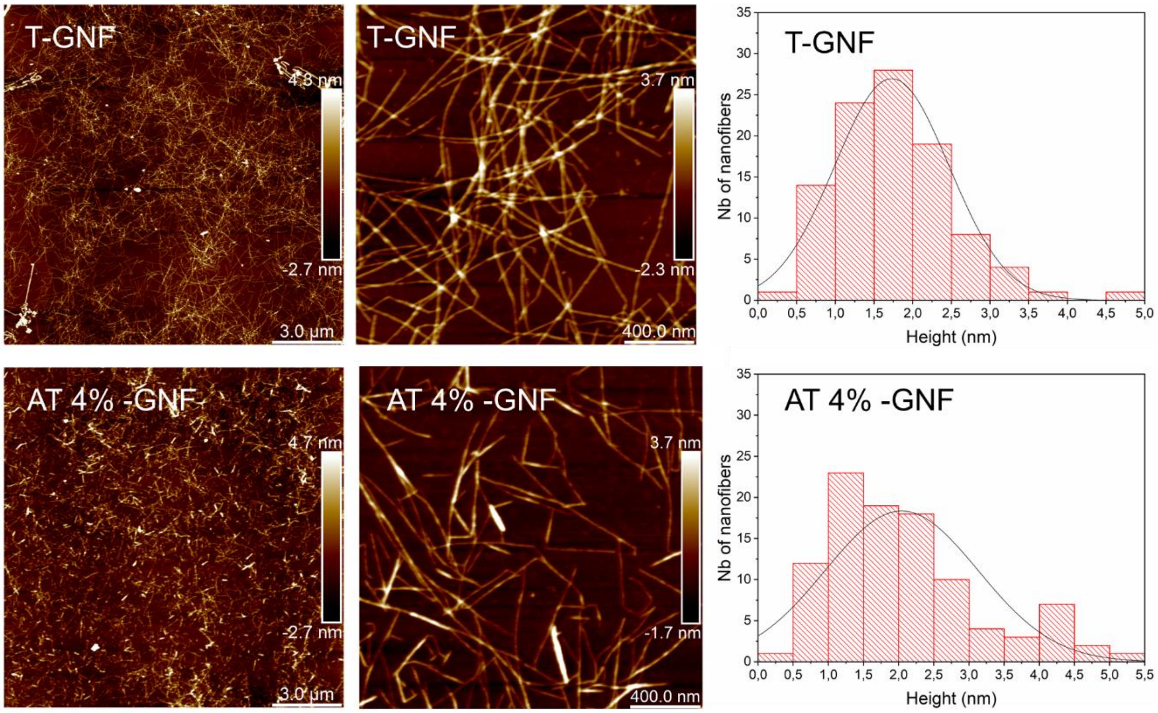

Figure S1. AFM height images and size distribution of the T-GNF and AT 4% - GNF, respectively.

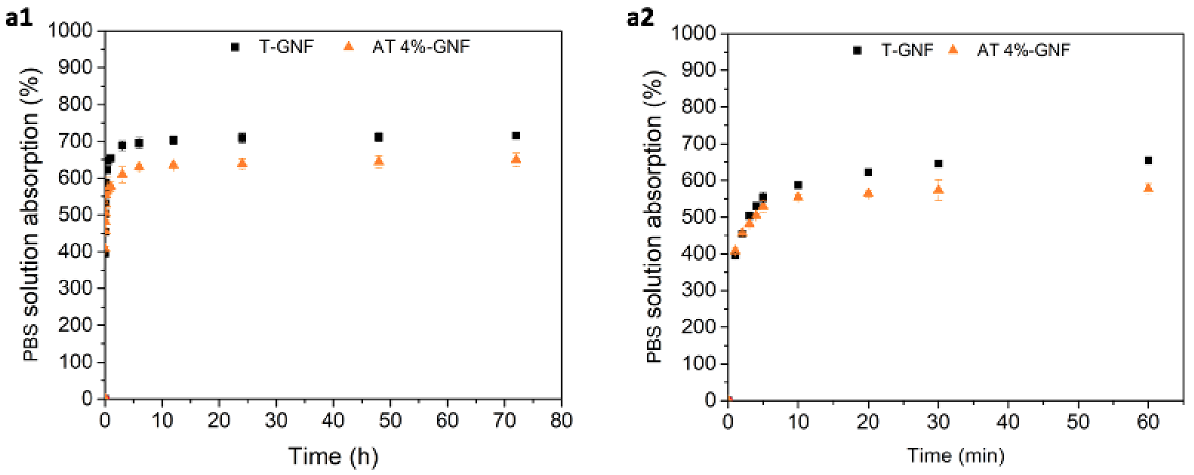

Figure S2. a1) Liquid absorption of the hydrogels (40 g m^{-2}) in PBS solution. a2) Expanded curves (first hour) of the liquid absorption of hydrogels in PBS solution.

Table S1. Hydrogel integrity in BSA solution.

Hydrogel	Days					
	1	5	10	15	20	40
T-GNF (%)	100	100.4 ± 0.8	101.7 ± 2.2	100.6 ± 3.4	102.1 ± 3.5	100.1 ± 0.7
4% AT-GNF (%)	100	102.4 ± 0.8	105.7 ± 4.6	112.1 ± 1.4	103.4 ± 1.9	102.2 ± 2.5

Table S2. Hydrogel integrity in PBS solution.

Hydrogel	Days					
	1	5	10	15	20	40
T-GNF (%)	100	100.7 ± 1.8	101.0 ± 0.7	100.5 ± 1.4	100.2 ± 0.4	100.7 ± 0.7
4% AT-GNF (%)	100	100.3 ± 1.0	100.6 ± 0.7	100.6 ± 0.7	100.3 ± 1.3	100.9 ± 0.5

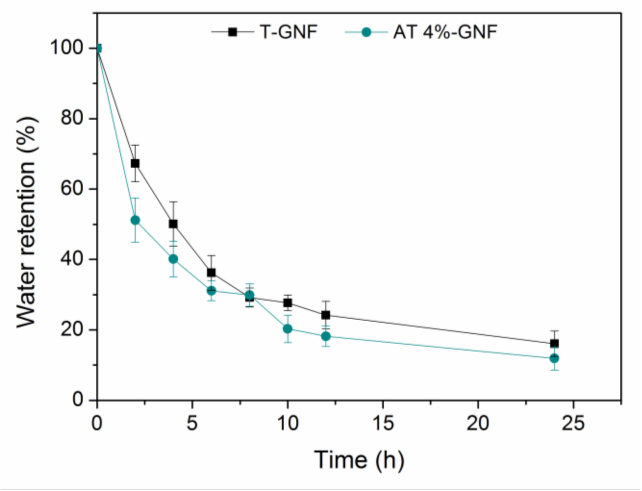

Figure S3. Water retention capacity of T-GNF and AT 4%-GNF hydrogels (40 g m^{-2}).

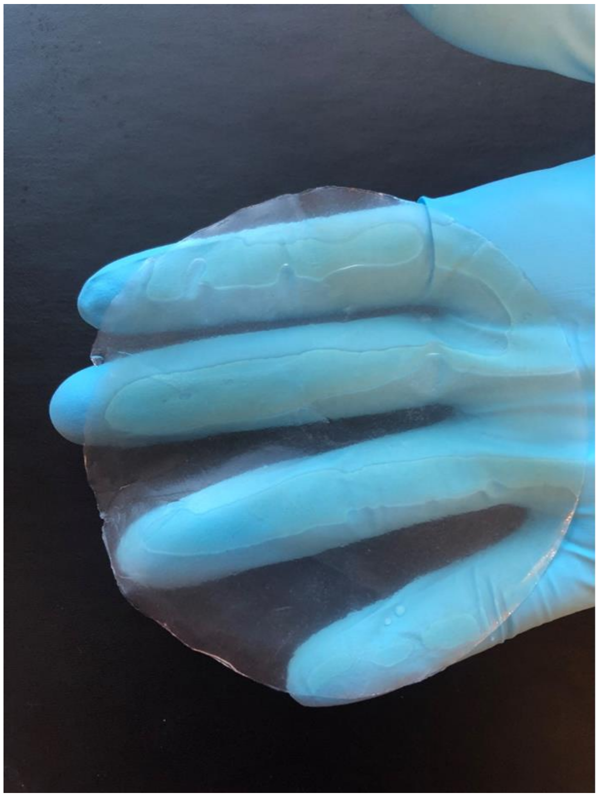

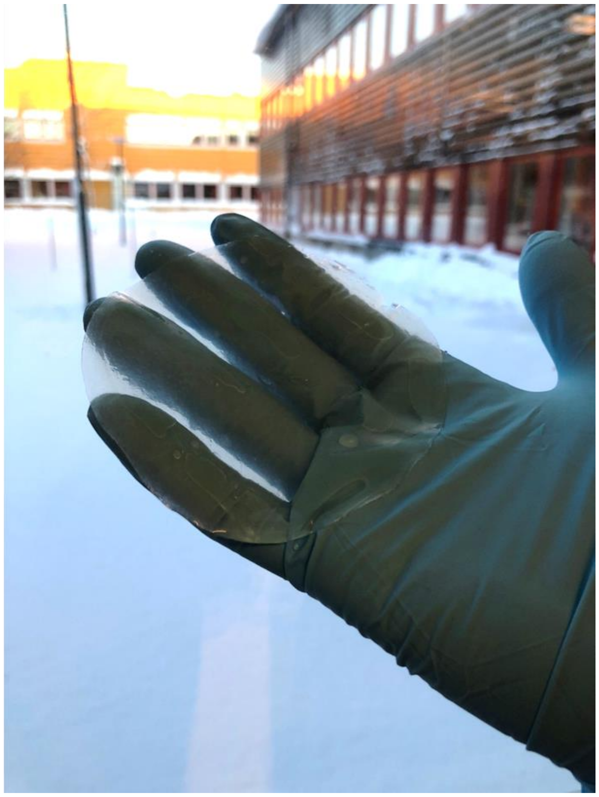

Figure S4. Photographs of the T-GNF hydrogels at 40 g m^{-2} after reaching the equilibrium state.

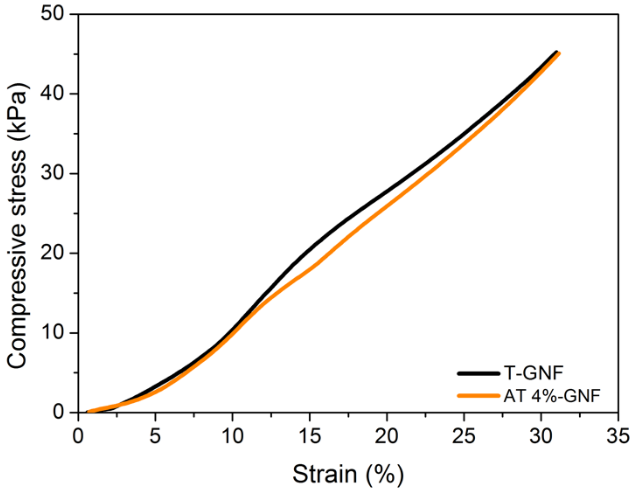

Figure S5. The representative compressive stress–strain curves enlarged at 30% strain.

Table S3. Mechanical properties of the hydrogels.

Hydrogel	Compressive Modulus (kPa)	Compressive strenght (kPa)	E-modulus (MPa)	Tensile strength (MPa)	Elongation at break (%)
T-GNF	77.2 (1.6)[a]	445.9 (2.1)[a]	15.3 (0.5)[a]	2.1 (0.2)[a]	25.1 (1.4)[a]
AT 4%-GNF	33.7 (4.7)[b]	210.5 (5.6)[b]	12.1 (0.1)[b]	1.6 (0.1)[b]	17.8 (1.0)[b]

*Average values with different superscript letters in the same column are significantly different at 5% significance level ($p < 0.05$) based on ANOVA test. Results are expressed as the average value (Standard Deviation).

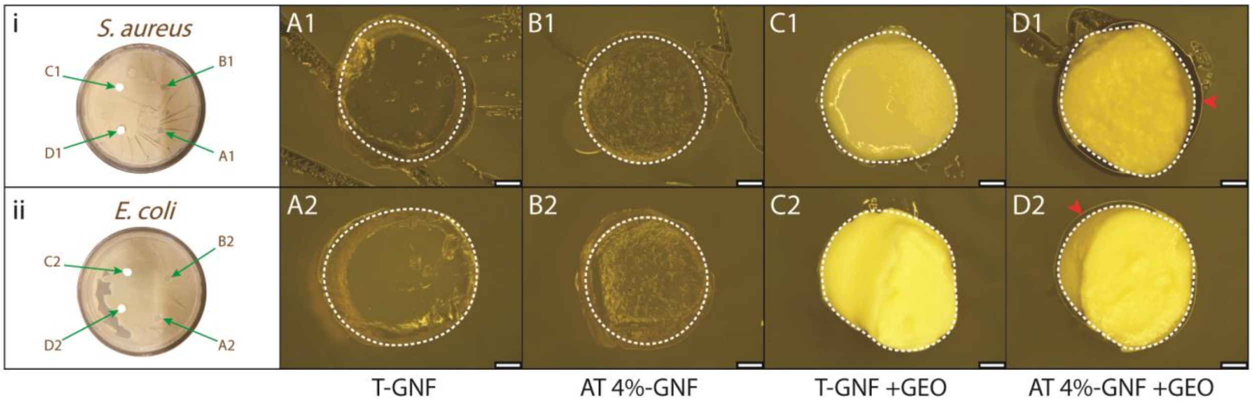

Figure S6. Overview images from microbiological assay for inhibition zones. Top row: i/1) *S. aureus* and bottom row ii/2) *E. coli*. A) T-GNF, B) AT 4%-GNF, C) T-GNF after functionalized with ginger essential oil (GEO), D) AT 4%-GNF with GEO. Red arrowheads indicate potential zone of inhibition. Dashed lines approximate hydrogel perimeters. Magnification 12.5x. (Scale bar: 1 mm).

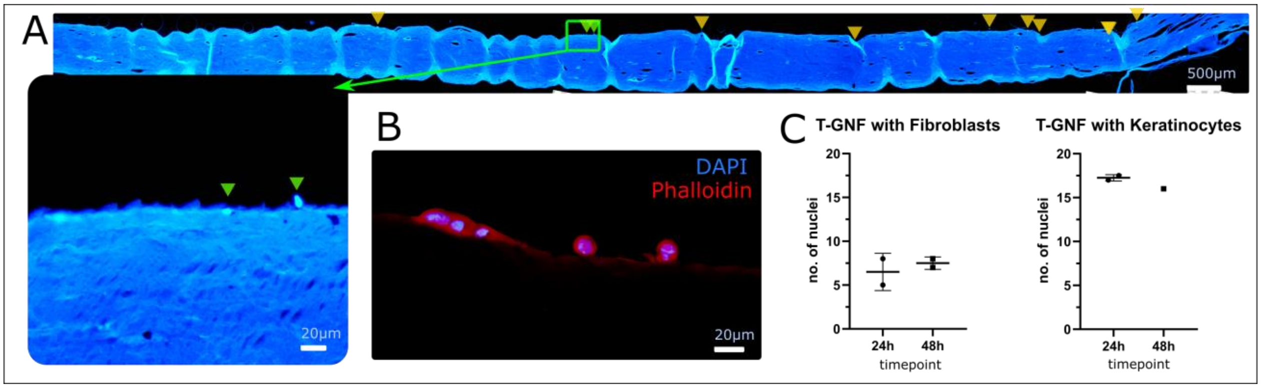

Figure S7. Investigation of cell attachment to the T-GNF hydrogel. A) acquired stitch at 10x magnification under UV-excitation along paraffin sectioned T-GNF hydrogel (~7 µm thick) with DAPI-labeled cells – inset: enlargement showing example of nuclei on the hydrogel surface (green arrowheads). B) Cells (keratinocytes shown) were labeled with DAPI and ALEXA546-conjugated Phalloidin. C) duplicate samples of fibroblasts and keratinocytes on T-GNF were quantified. Arrowheads indicate DAPI-stained nuclei.

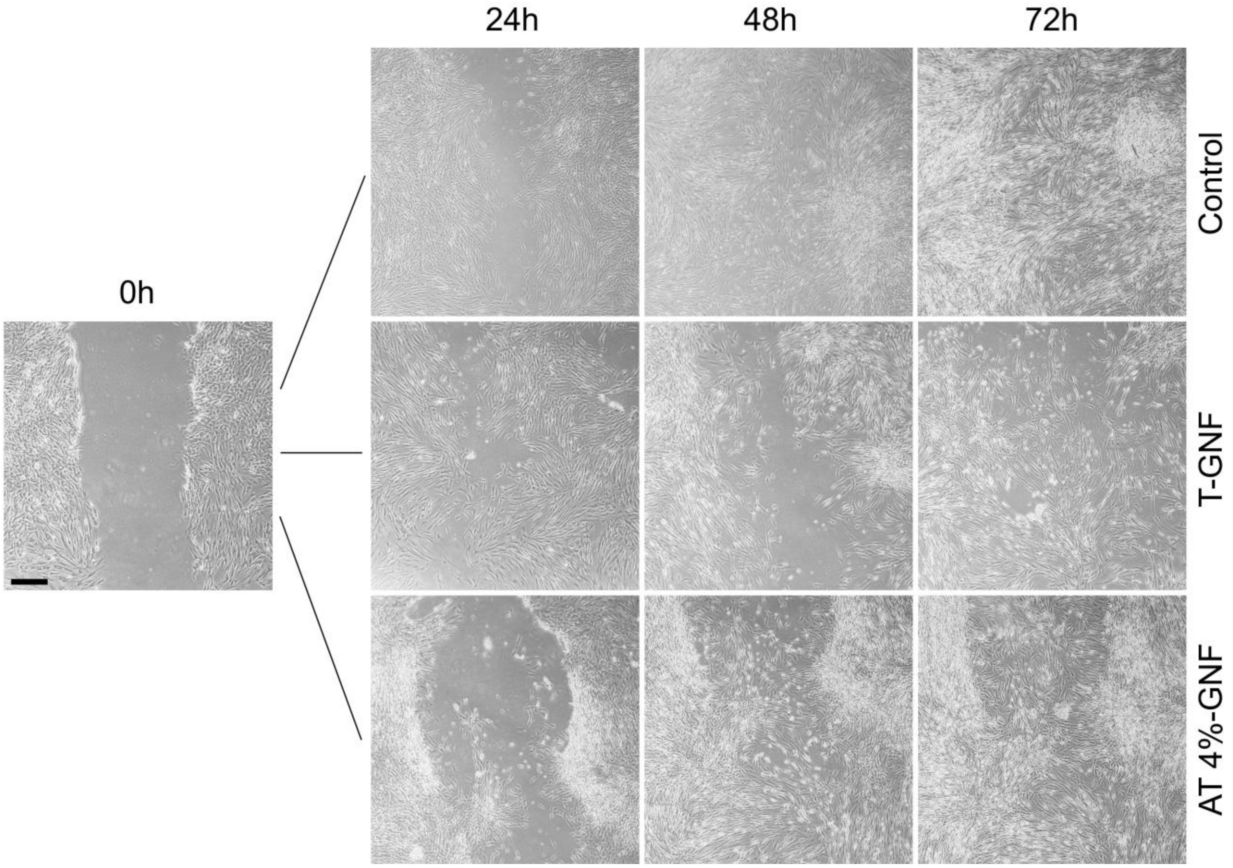

Figure S8. Images from scratch assay showing migration of fibroblasts at different time points. Scale bar corresponds to ~100µm.

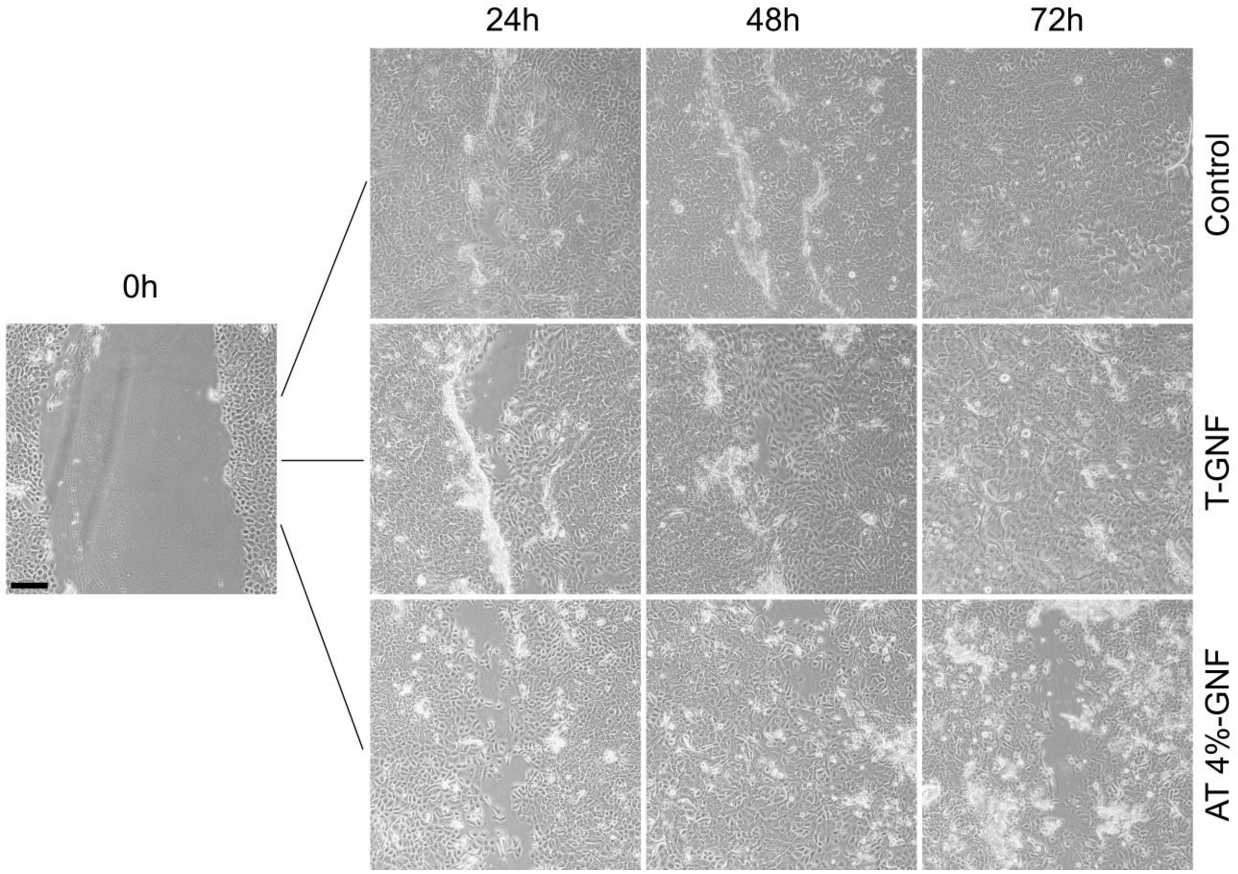

Figure S9. Images from scratch assay showing migration of keratinocytes at different time points. Scale bar corresponds to ~50μm.
